# Myocarditis

**Published:** 1907-08-31

**Authors:** 


### Diseases of Children.

### MYOCARDITIS.

At the provincial meeting of the Society for the Study of Disease in Children last June there was a discussion on acute rheumatism in childhood. Although the frequency of cardiac complications was insisted on, comparatively little attention was directed to myocarditis and its treatment. The heart muscle is peculiarly susceptible to the influence of the rheumatic poison in early life, and actual myocarditis may occur independently, as well as in conjunction with endocarditis or pericarditis. Its development in the absence of a cardiac murmur is of the utmost importance, for in such a case it is liable to be overlooked, or the secondary dilatation of the heart is put down to the anæmia and debility.

Degeneration of the cardiac muscle occurs from the effect of toxins, *e.g.* those of influenza, diphtheria, and other infective disorders; or may follow the cloudy swelling which is often seen as the result of fever. Myocarditis may be acute or chronic, interstitial or parenchymatous, diffuse or circumscribed. The parenchymatous type is the one most often seen in the young, and it is commonly due to the rheumatic poison.

A few years ago I saw in consultation a girl, aged eleven years, who for two weeks had been suffering from fever, sleepiness, impaired appetite, slight cough, and restless nights. The temperature ranged from 99° F. in the morning to 100° F. at night. She was supposed to have early phthisis. There was no family or past history of rheumatism, but a month previously she had had an attack of tonsillitis. On examination the heart was found much dilated, but there was no murmur. A diagnosis of rheumatic myocarditis was made and recovery ensued after two months' treatment by salicylates and rest. I saw her again two years later. The apex of the heart was in the nipple line and there were no cardiac symptoms. The child had had rheumatic joint swelling during the interval.

### Diagnosis and Treatment.

Diagnosis is difficult, unless there is definite evidence of rheumatism. It may be impossible to differentiate the inflammatory affection and its results from the dilatation due to loss of tone of the cardiac muscle or degenerative changes. When, however, dilatation is found in the course of rheumatic fever, or in the absence of any distinct exciting cause in a child, it should be regarded as due to rheumatic myocarditis and treated accordingly. The essentials of treatment are the same as in other forms of dilatation, namely, complete rest and careful feeding, with the avoidance of foods liable to cause indigestion and of all forms of excitement. Salicylates and alkalies are of value in the rheumatic type, but in dilatation due to other causes they may be disadvantageous. In severe cases oxygen inhalations and even venesection may be needed. Stimulants are frequently required. The prognosis depends on the causation, the extent of the dilatation, and on the treatment. Provided the affection is rheumatic, that the child is seen before the dilatation is extreme, and that the patient is kept completely at rest in bed for six to eight weeks or more, recovery is probable. The outlook is worse if there is endocarditis present, and still more serious if there is pericarditis or adhesion of the pericardium. In the latter event the pulse remains persistently small and unduly frequent, the child is short of breath on slight exertion, and the appetite is impaired. Gain in weight and decrease in the pulse rate are good signs.

The extent of the dilatation is to some extent of value in diagnosis. If it is limited to the left ventricle it is most likely to be due to diphtheria. In such cases the more the dilatation extends beyond the nipple line the greater is the danger. Sudden death may occur at any time, even when the heart is only moderately dilated. It is more apt to result from a degenerated diphtheritic heart than from rheumatic myocarditis.

### Further Points in Treatment.

Rest is of vital importance. It is easy to enforce if the child is seriously ill. It is difficult to maintain during convalescence and in mild degrees of the affection. Where possible, a trained nurse should be obtained during the acute stages. The child must be kept completely lying down and not allowed to sit up under any circumstances. It must not be excited, must not be allowed visitors, and must be kept as quiet as possible. The greater the degree of dilatation, the longer must this rigid rest be insisted upon. In no case is it advisable to limit it to less than a month. The pulse rate forms a fairly reliable guide. The pulse should be counted every four hours and charted in the same way as the temperature is recorded. When it has come down to the normal rate, the child may be propped up in bed for a short time, and the effect noted. If the dilatation has been severe, passive and opposed movements must be made use of for a few days before allowing greater muscular effort. Gradually the amount of exertion may be increased, provided no ill effects ensue. Later, the child may be allowed to get up, walk about the room, down stairs, on the level, slowly up stairs, and finally up-hill. Bicycling on the level is permissible. After six months to a year games, dancing, and gymnastic exercises can be indulged in, provided they do not involve competition and the child can stop whenever it likes. After a serious attack, precautions against over-exertion must be adopted for at least a year. The evil effects of strain are indicated by shortness of breath, inability to take food, sleeplessness, and indigestion. On the other hand there is no necessity to allow the child to become a chronic invalid. Few cases need the tactful management of the doctor more than these do. Often it is the parents who require management rather than the child. Never forget that permanent damage may result from insufficient care, and that a prolongation of convalescence is a delay of little importance in childhood.

